# Tools for T-RFLP data analysis using Excel

**DOI:** 10.1186/s12859-014-0361-7

**Published:** 2014-11-08

**Authors:** Nils Johan Fredriksson, Malte Hermansson, Britt-Marie Wilén

**Affiliations:** Department of Medical Biochemistry and Cell Biology, Institute of Biomedicine, Sahlgrenska Academy, University of Gothenburg, Gothenburg, Sweden; Department of Chemistry and Molecular Biology, University of Gothenburg, Gothenburg, Sweden; Department of Civil and Environmental Engineering, Water Environment Technology, Chalmers University of Technology, Gothenburg, Sweden

**Keywords:** Terminal restriction fragment length polymorphism data analysis, Microbial ecology, DNA-fingerprinting

## Abstract

**Background:**

Terminal restriction fragment length polymorphism (T-RFLP) analysis is a DNA-fingerprinting method that can be used for comparisons of the microbial community composition in a large number of samples. There is no consensus on how T-RFLP data should be treated and analyzed before comparisons between samples are made, and several different approaches have been proposed in the literature. The analysis of T-RFLP data can be cumbersome and time-consuming, and for large datasets manual data analysis is not feasible. The currently available tools for automated T-RFLP analysis, although valuable, offer little flexibility, and few, if any, options regarding what methods to use. To enable comparisons and combinations of different data treatment methods an analysis template and an extensive collection of macros for T-RFLP data analysis using Microsoft Excel were developed.

**Results:**

The *Tools for T-RFLP data analysis* template provides procedures for the analysis of large T-RFLP datasets including application of a noise baseline threshold and setting of the analysis range, normalization and alignment of replicate profiles, generation of consensus profiles, normalization and alignment of consensus profiles and final analysis of the samples including calculation of association coefficients and diversity index. The procedures are designed so that in all analysis steps, from the initial preparation of the data to the final comparison of the samples, there are various different options available. The parameters regarding analysis range, noise baseline, T-RF alignment and generation of consensus profiles are all given by the user and several different methods are available for normalization of the T-RF profiles. In each step, the user can also choose to base the calculations on either peak height data or peak area data.

**Conclusions:**

The *Tools for T-RFLP data analysis* template enables an objective and flexible analysis of large T-RFLP datasets in a widely used spreadsheet application.

**Electronic supplementary material:**

The online version of this article (doi:10.1186/s12859-014-0361-7) contains supplementary material, which is available to authorized users.

## Background

Terminal restriction fragment length polymorphism (T-RFLP) analysis is a DNA-fingerprinting method that can be used for comparison of the microbial community composition in a large number of samples [1]. In T-RFLP analysis a gene, or a section of a gene, is amplified by PCR with at least one of the primers labeled with a fluorescent marker. The amplified genes are cut into fragments by a restriction enzyme and the resulting restriction fragments are separated by size using polyacrylamide or capillary gel electrophoresis. The terminal restriction fragments (T-RFs), which are labeled with the fluorescent marker, are detected by an automated DNA sequencer. Each T-RF results in a peak in an electropherogram, where the peak height and area are determined by the strength of the fluorescent signal which in turn is determined by the amount of the T-RF in the sample, i.e. the number of genes with that particular T-RF length. By including reference fragments of known lengths, a size standard, the lengths of the T-RFs can be derived from the migration time through the gel. The set of T-RFs of different lengths obtained from a sample is referred to as a T-RF profile and can be regarded as a DNA-fingerprint of the microbial community in the sample. The length of a T-RF is defined by the presence and position of restriction enzyme recognition sites and different gene sequences therefore generate T-RFs of different lengths. As the presence of a T-RF of a certain length indicates the presence of certain gene sequences, differences in microbial community composition between samples can be inferred by differences in detected T-RFs in the T-RF profiles of the samples. To what extent the differences in microbial community composition between two samples are detected depends on the choice of primers and enzymes. To evaluate the suitability of different primers and enzymes for T-RFLP analysis several tools are available [2-4].

The analysis of T-RFLP data can be cumbersome and time-consuming, and for large datasets manual data analysis is not feasible. To ensure that the observed differences in T-RF profiles are due to differences in community composition and not introduced by the sample handling or data processing, there are three important steps in the data analysis: (1) the removal of noise or false peaks, (2) the alignment of the T-RFs and (3) the standardization, or normalization, of the T-RF profiles. Different approaches for removal of noise in the literature include the application of a high peak detection threshold [5], only considering T-RFs that are reproducible in several replicates [6] and statistical methods [7]. During alignment of the T-RF profiles it is determined which T-RFs that are the same in two or more samples. Estimation of the T-RF sizes is not exact and can vary between samples. Because of this, alignment can be both difficult and time-consuming, especially when analyzing large datasets. The alignment can be done manually (e.g. [8,9]) but several non-subjective alignment methods have also been presented (e.g. [6,7,10]). Normalization of T-RF profiles aims at removing differences in T-RF profiles due to variations in the amount of DNA that was loaded on the gel and several different approaches have been proposed (e.g. [6,7,9,11-14]). Another aspect of T-RFLP data analysis is whether to base the analyses on peak height or peak area data. The argument for using peak area data is that peak heights decrease with increasing migration times in the gel and the abundances of long T-RFs will therefore be underestimated if peak height data is used [15]. However, the calculation of peak areas may be skewed by overlapping peaks while peak height calculations are not. Furthermore, peak height data has been shown to better reflect the ratios of defined sample concentrations than peak area data [16]. In summary, there is no absolute consensus on how T-RFLP data should be treated and analyzed before comparisons between samples are made.

A number of different tools have been made available for the automated analysis of T-RFLP data, all with their own merits. *T-Align* [10] can be used for alignment of duplicates, generation of consensus profiles and alignment of the consensus profiles. However, the number of replicates is fixed to two, and there are no options for normalization. *T-REX* [17] has two options for alignment: either simply replacing the observed T-RF sizes with the value of the nearest integer or using an adaptation of the *T-Align* alignment method. However, there is only one option for noise removal, the method by Abdo *et al*. [7] and there is no flexibility in the generation of consensus profiles. The *T-RFLP Stats* scripts by Abdo *et al*. [7] only perform the methods described in the paper without any other options. None of the available tools enable easy comparison of different normalization strategies. They also lack options for consensus profile generation and tools for the evaluation of the T-RF alignment. To meet this need, the collection of Visual Basic macros and the analysis template presented here, from now on referred to as the *Tools for T-RFLP data analysis*, were developed. The procedures allow for automated normalization and alignment of replicate profiles, creation of consensus profiles from replicate profiles and normalization and alignment of the consensus profiles. Included are also procedures to evaluate the accuracy of the resulting alignments, to calculate association coefficients between T-RF profiles and a diversity index. The macros and the analysis template sheet are designed so that adjustments of the parameters for noise baseline, T-RF analysis range, normalization and alignment can easily be made. In addition, all analyses can be based on either peak height data or peak area data.

## Implementation

The *Tools for T-RFLP data analysis* is provided as an Excel file (Additional file 1: Tools for T-RFLP data analysis.xlsm), which includes the necessary Visual Basic macros. All calculations and storage of data in intermediate and final steps are done in the spreadsheets. This may not be the fastest or most efficient way, but it allows the user fast access to the data and enables easy additional manipulation of the data in a, for many researchers familiar, spreadsheet application.

Although Excel is software written for the Windows operating system, it can also be used on computers with other operating systems through the use of virtualizers, which enables more than one operating system to be run at the same time.

### Input data

The input T-RFLP data must be given in the sheet ”Input Data”. The data must be in the format as given by the example in Table 1 or in the sheet ”Input Data Example”. This format is easily obtained directly from programs such as GeneMapper (Applied Biosystems), which is common for the analysis of raw data.

**Table 1:** **Input data format with example data**

**Sample Name**	**Sample 1.1**	**Sample 1.2**	**Sample 2.1**	**Sample 2.2**	**Sample 3.1**	**Sample 3.2**
**T-RF 1**	1	1	1	1	1	1
**T-RF 2**	1	1	1	1	1	1
**T-RF 3**	0	1	1	1	0	0
**Size 1**	167.78	167.75	167.78	167.87	167.78	167.88
**Size 2**	300	299.92	300	299.84	300	299.92
**Size 3**		478.02	478.24	478.23		
**Height 1**	900	578	900	426	900	362
**Height 2**	640	434	640	331	640	278
**Height 3**		720	966	488		
**Area 1**	10798	7164	10798	5293	10798	4393
**Area 2**	7376	4869	7376	3807	7376	3129
**Area 3**		11992	15617	7958		

An example of six samples with at most three T-RFs each. Columns are samples and rows are sample names, presence or absence values (0 or 1), T-RF sizes, peak heights and peak areas.

### Input parameters

Initial parameters regarding data range, alignment, normalization, consensus profile generation and number of profiles, samples and replicates must be given in the sheet “Input”. The parameters and examples of values are given in Table 2. Parts of the results of the analyses will also be written in the ”Input” sheet.

**Table 2:** **Required input parameters with example values**

**Parameter**	**Value**
**1. Total number of profiles**	6
**2. Number of samples**	3
**3. Number of replicates per sample**	2
**4. Number of T-RFs**	3
**5. Sample names on row**	1
**6. Consider T-RF if present in X replicates. X=**	2
**7. Align T-RFs with a size difference shorter than Y bases. Y=**	1
**8. Add T-RFs less than Z bases longer than the average size of the alignment. Z=**	0.5
**9. Peak height detection threshold. PDT =**	50
**10. Lower T-RF Size Limit**	50
**11. Upper T-RF Size Limit**	1020
**12. Fixed percentage threshold. FPT=**	0.01

Parameters 1–5 and 9–11 are used for the initial preparation of the data. Parameters 1–4 are also used by procedures that need the number of samples or replicates to be specified. Parameter 6 is used for the generation of consensus profiles. Parameters 7 and 8 are used in the alignment of T-RF profiles. Parameter 12 is used for normalization with the fixed percentage threshold procedure.

### Analysis

**Figure 1:**
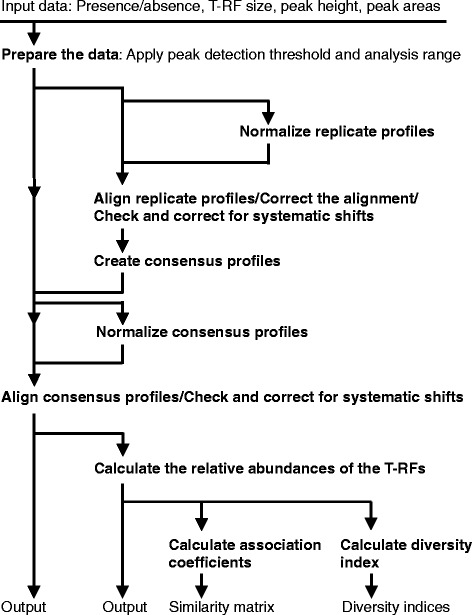
**Order and possible combinations of analysis steps.** The analysis steps outlined in the figure correspond to the headings in Table 3.

The different steps of the data analysis are outlined in the sheet ”Analysis protocol - All tools” in the analysis template (Additional file 1) and in Table 3. Figure 1 shows the order and possible combinations of the analysis steps. Rather than having one procedure performing all steps the user must run the macros for the different steps. This allows for flexibility regarding what steps to include in the analysis. Between the steps the user also has the option to change the data that is going to be used, by copying the relevant data to the sheet “InData”. This allows for flexibility regarding what data to use in each step of the analysis. The corresponding procedures and input and output data sheets for the different analysis steps are shown in Table 3.

**Table 3:** **Analysis steps and the corresponding procedures**

**Step**	**Macro name**	**Input sheet**	**Output sheet**
**Prepare the data**			
1	A_Prepare_Alignment	Input Data	AppliedPDTandAnalysisRange
			TempWorking
			InData
			TempInData
**Normalize replicate profiles**			
**2**	Relevant data is copied to InData	AppliedPDTandAnalysisRange	InData
**3 alt. 1**	B_Normalize_replicates_TFNAreas	InData	NormalizedReplicatesAreas
**3 alt. 2**	B_Normalize_replicates_TFNAreas_LT	InData	NormalizedReplicatesAreasLT
**3 alt. 3**	B_Normalize_replicates_TFNHeights	InData	NormalizedReplicatesHeights
**3 alt. 4**	B_Normalize_all_FPTAreas	InData	AllNormalizedFPTAreas
**3 alt. 5**	B_Normalize_all_FPTHeights	InData	AllNormalizedFPTHeights
**Align replicate profiles**			
**4**	Relevant data is copied to InData	E.g. NormalizedReplicatesHeights	InData
**5**	C_Align_replicate_profiles	InData	ReplicateSamplesAligned
**Correct the alignment**			
**6**	D_FindAndCorrect-AmbiguousAlignments	InData	CorrectedRepSamplesAligned
**Check and correct for systematic differences in size estimation between replicates**			
**7**	Relevant data is copied to InData	E.g. CorrectedRepSamplesAligned	InData
**8**	D_CheckReplicateSystematicShifts	InData	RepSystematicShiftCheck
			RepSystematicShiftMatrix
**9**	F_ShiftCorrectedReplicateSizes	InData	ReplicateRelativeSizes
			NewReplicateSizes
			NewReplicateSizesAligned
**Create consensus profiles**			
**10**	Relevant data is copied to InData	E.g. CorrectedRepSamplesAligned	InData
**11 alt. 1**	G_RepConsensus_Average	InData	RepConsensus
**11 alt. 2**	G_RepConsensus_Sum	InData	RepConsensusSum
**Normalize consensus profiles**			
**12**	Relevant data is copied to InData	E.g. RepConsensus	InData
**13 alt. 1**	H_Normalize_all_TFNAreas	InData	AllNormalizedTFNAreas
**13 alt. 2**	H_Normalize_all_TFNHeights	InData	AllNormalizedTFNHeights
**13 alt. 3**	B_Normalize_all_FPTAreas	InData	AllNormalizedFPTAreas
**13 alt. 4**	B_Normalize_all_FPTHeights	InData	AllNormalizedFPTHeights
**Align consensus profiles**			
**14**	Relevant data is copied to InData	E.g. AllNormalizedTFNHeights	InData
**15**	I_Align_consensus_profiles	InData	AlignedConsensus
**16**	J_Check_AlignedConsensus	AlignedConsensus	AlignedConsensus
**Check and correct for systematic differences in size estimation between consensus profiles**			
**17**	Relevant data is copied to InData	E.g. AlignedConsensus	InData
**18**	K_CheckConsensusSystematicShifts	InData	ConsSystematicShiftCheck
			ConsSystematicShiftMatrix
**19**	L_Shift_corrected_consensus_sizes	InData	ConsensusRelativeSizes
			ConsRelativeSizesStDevs
			AlignedConsShiftCorrSizes
**Calculate the relative abundances of the T-RFs**			
**20**	Relevant data is copied to InData	E.g. AlignedConsensus	InData
**21 alt. 1**	M_Relative_abundance_Areas	InData	RelativeAbundanceAreas
**21 alt. 2**	M_Relative_abundance_Heights	InData	RelativeAbundanceHeights
**Calculate association coefficients**			
**22**	Relevant data is copied to InData	E.g. RelativeAbundanceHeights	InData
**23**	N_BrayCurtis_matrix	InData	BrayCurtisMatrix
**24**	O_JaccardCoeff_matrix	InData	JaccardCoeffMatrix
**Calculate diversity index**			
**25**	Relevant data is copied to InData	E.g. RelativeAbundanceHeights	InData
**26**	P_ShannonIndex	InData	Shannon Index

### Documentation

As the typical analysis of T-RFLP data includes several different steps involving a number of different parameters it is good practice to document the analysis. An analysis history including information about input data, applied procedure and output data is automatically created in the sheet “Run history”.

### Restrictions and limitations

The maximum number of fragments is 1500 per profile. However, this number can be changed by changing the appropriate numbers in the code for the macros.

If the dataset contains sample replicates it is assumed that all samples have the same number of replicates.

The procedures do not check that the given data has the right format. If the data is formatted the wrong way or parameters are not given an error message will be given by Excel.

If a procedure is run more than once an error message will appear that says: “Cannot rename a sheet to the same name as another sheet…”. This is because the procedure tries to create a new sheet with the same name as a sheet which was created during the first run. To run a procedure more than once the user must delete or rename the sheets that were created by the procedure the first time.

## Description of procedures

### Preparation of the data

#### Macro: A_Prepare_Alignment

This procedure uses the data in the sheet “Input Data” and the parameters in the sheet “Input”. Note that the value 0 and blank cells are treated indistinctively. The procedure applies the peak detection threshold and T-RF length restrictions given in the “Input” sheet and copies sample names, peak sizes, heights and areas to a new sheet called ”AppliedPDTandAnalysisRange”. Three new sheets that are used in the following analyses are also created: ”InData”, “TempInData” and “TempWorking”. The maximum observed number of T-RFs in a profile after application of threshold and T-RF length restrictions is written in the “Input” sheet. The data in the sheet ”AppliedPDTandAnalysisRange” is copied to the sheet “InData” for further analysis.

### Normalization of replicate profiles

There are five options for the normalization of replicate profiles.

Three of the available procedures use the total fluorescence normalization (TFN) procedure described by Dunbar *et al*. [6]. This procedure normalizes the profiles so that all profiles will have the same, or similar, total fluorescence. The total fluorescence is defined as either the sum of all peak heights or peak areas in a profile. All T-RFs of a profile with a total fluorescence, TF, higher than the lowest total fluorescence in the dataset, TFmin, are multiplied with a factor TFmin/TF. T-RFs that fall below a defined threshold after the multiplication are removed and a new TF is calculated with the remaining T-RFs. The new TF is compared with TFmin once again and the procedure is reiterated until the new TF is equal to TFmin. If the new TF oscillates between two states, higher than and lower than TFmin, due to the inclusion or exclusion of a T-RF close to the threshold, the profile is calculated taking the average of the two states.

The two remaining procedures use an approach where all T-RFs with a relative abundance below a fixed percentage threshold (FPT) are removed. The FPT must be given in the “Input” sheet. The relative abundance of a T-RF is the peak height (or area) divided by the total fluorescence, i.e. the sum of all peak heights (or areas) in that profile. The FPT approach has previously been described and applied by Li *et al*. using peak areas [14].

#### Macro: B_Normalize_replicates_TFNAreas

TF is defined as the sum of all peak areas. The minimum allowed peak area is the minimum observed peak area among all profiles in the dataset, not just among the replicates that are normalized. The procedure uses the sheet “TempWorking” for calculations, the data in the sheet “InData” and the parameters in the sheet “Input”. Creates a new sheet called ”NormalizedReplicatesTFNAreas”. The data in the sheet ”NormalizedReplicatesTFNAreas” is copied to the sheet “InData” for further analysis.

#### Macro: B_Normalize_replicates_TFNAreas_LT

TF is defined as the sum of all peak areas. The minimum allowed peak area is the minimum observed peak area among the replicates that are normalized. This procedure is equivalent to the normalization procedure described by Kaplan *et al*. [12]. The procedure uses the sheet “TempWorking” for calculations, the data in the sheet “InData” and the parameters in the sheet “Input”. Creates a new sheet called ”NormalizedReplicatesTFNAreasLT”. The data in the sheet ”NormalizedReplicatesTFNAreasLT” is copied to the sheet “InData” for further analysis.

#### Macro: B_Normalize_replicates_TFNHeights

Total fluorescence is defined as the sum of all peak heights. This procedure is identical to the procedure by Dunbar *et al*. [6]. The minimum allowed peak height is the peak detection threshold given in the “Input” sheet. The procedure uses the sheet “TempWorking” for calculations, the data in the sheet “InData” and the parameters in the sheet “Input”. Creates a new sheet called ”NormalizedReplicatesTFNHeights”. The data in the sheet ”NormalizedReplicatesTFNHeights” is copied to the sheet “InData” for further analysis.

#### Macro: B_Normalize_all_FPTAreas

This procedure normalizes all samples using a fixed percentage threshold given in the “Input” sheet. As each profile is normalized independent of the other profiles in the dataset the same procedure can be applied for both replicate and consensus profiles. The relative abundances of all T-RFs are calculated using peak areas and values are only copied for T-RFs with a relative abundance above the threshold. The procedure uses the data in “InData” and creates a new sheet called ”AllNormalizedFPTAreas”. The data in the sheet ”AllNormalizedFPTAreas” is copied to the sheet “InData” for further analysis.

#### Macro: B_Normalize_all_FPTHeights

This procedure normalizes all samples using a fixed percentage threshold given in the “Input” sheet. As each profile is normalized independent of the other profiles in the dataset the same procedure can be applied for both replicate and consensus profiles. The relative abundances of all T-RFs are calculated using peak heights and values are only copied for T-RFs with a relative abundance above the threshold. The procedure uses the data in “InData” and creates a new sheet called ”AllNormalizedFPTHeights”. The data in the sheet ”AllNormalizedFPTHeights” is copied to the sheet “InData” for further analysis.

### Alignment of replicate profiles

The replicate profiles are aligned using the moving average procedure described by Smith *et al*. for *T-Align* [10]. The shortest T-RF of all profiles is identified and placed in an alignment bin. All other T-RFs that are at most Y bases longer than the first T-RF are also included in the alignment bin. The average length of all T-RFs within the alignment bin is then calculated and any additional T-RFs that are at most Z bases longer than the average length of the bin are also included. If a T-RF is added a new average length is calculated and a new search is done to see if more T-RFs are now within the distance Z from the new average and thus should be added. If no additional T-RFs are added a new alignment bin is created and the process starts over with the remaining T-RFs, identifying the shortest T-RF of all profiles that is not already in an alignment bin. A T-RF profile is only allowed to have one T-RF in each alignment bin. The parameters Y and Z must be given by the user in the “Input” sheet.

The resulting alignment is not always accurate. If one replicate has a T-RF with a size in between two T-RFs that both are within the alignment range in the other replicates, the shortest T-RFs will always be aligned together, even if the longest T-RFs are more similar in size (Table 4). This happens because the alignment process works from shorter T-RFs to longer T-RFs, without checking if alternative ways of binning the T-RFs are more accurate. For duplicates this can easily be resolved, by binning the T-RFs that are most similar in size (Table 4). For more than two replicates the same correction procedure can be applied but what the correct alignment really is, is not always as easily determined (Table 5).

**Table 4:** **Examples of automatic and corrected alignment of two profiles with four T-RFs**

**Automatic alignment**	**Sample 1**	**Sample 2**
**T-RF 1**	207.43	208.26
**T-RF 2**	208.52	0
**T-RF 3**	433.69	432.71
**T-RF 4**	0	433.84
**Corrected alignment**	**Sample 1**	**Sample 2**
**T-RF 1**	207.43	0
**T-RF 2**	208.52	208.26
**T-RF 3**	0	432.71
**T-RF 4**	433.69	433.84

The given numbers are the T-RF sizes in bases.

**Table 5:** **An example of automatic and corrected alignment of four profiles with two T-RFs**

**Automatic alignment**	**Sample 1**	**Sample 2**	**Sample 3**	**Sample 4**
**T-RF 1**	254.11	253.81	0	254.66
**T-RF 2**	0	0	254.87	0
**Corrected alignment**	**Sample 1**	**Sample 2**	**Sample 3**	**Sample 4**
**T-RF 1**	254.11	253.81	0	0
**T-RF 2**	0	0	254.87	254.66

The given numbers are the T-RF sizes in bases.

#### Macro: C_Align_replicate_profiles

This procedure aligns the replicate profiles using the moving average procedure as described above. The procedure uses the sheet “TempWorking” for calculations, the data in the sheet “InData” and the parameters in the sheet “Input”. Creates a new sheet called ”ReplicateSamplesAligned”. The number of alignment bins after alignment is written in the “Input” sheet. The data in the sheet ”ReplicateSamplesAligned” is copied to the sheet “InData” for further analysis.

#### Macro: D_FindAndCorrectAmbiguousAlignments

This procedure identifies T-RFs that are within the alignment range Y of both a shorter and a longer T-RF in the other replicates. The T-RFs are then aligned with the T-RF that is closest in size and size height and area data are adjusted accordingly. The data from ”ReplicateSamplesAligned” is copied and a new sheet called ”CorrectedRepSamplesAligned” is created. Cells with T-RFs that are within the alignment range of T-RFs in another alignment bin are highlighted in yellow and cells with T-RFs that have been changed to new alignment bins are framed. The number of possibly ambiguous T-RFs and the number of corrected alignments are written in the “Input sheet”. The data in the sheet ”CorrectedRepSamplesAligned” is copied to the sheet “InData” for further analysis.

### Detection and correction of systematic differences in size estimation between replicates

There are always variations in the size estimation of the T-RFs, even between subsequent loadings of the same sample, and this variation can cause errors in the alignment of the T-RFs. The following procedures first checks if the differences in T-RF sizes between two replicates are due to a systematic shift, i.e. if all T-RFs in one of the replicates are shorter than all T-RFs in the other. The systematic shift is then corrected for and new T-RF sizes are calculated and the profiles are re-aligned. Finally, the new alignment is compared with the original alignment and T-RFs that are binned differently in the two alignments are identified. The calculation of new T-RF sizes and re-alignment of the profiles is done for all replicates, regardless if there was a systematic shift in T-RF sizes between them. If the comparison with the alignment of shift-corrected T-RFs shows that the alignment of the replicate profiles needs to be corrected, the correction must be done manually, changing the corresponding size, height and area data.

#### Macro: E_CheckReplicateSystematicShifts

In this procedure pairwise comparisons are made between all replicates of the same sample. The sizes of the T-RFs in all alignment bins of the two replicates are compared. If all T-RFs in one of the replicates are shorter than all T-RFs in the other, there is a systematic shift in size estimation between the two. The procedure uses the data from the sheet “InData” and “Input” and creates new sheets called ”RepSystematicShiftCheck” and ”RepSystematicShiftMatrix”. In ”RepSystematicShiftCheck” the result of all T-RF size comparisons are given and in ”RepSystematicShiftMatrix” there is a matrix showing which profiles that display a systematic shift towards one another.

#### Macro: F_ShiftCorrectedReplicateSizes

This procedure calls the procedures F1 to F4, described below, for calculation and analysis of systematic shift corrected T-RF sizes.

#### Macro: F1_RelativeReplicateSizes

This procedure calculates new T-RF sizes using a T-RF common to all replicates as a reference which is set to size 0. Relative sizes, i.e. the difference in size between a T-RF and the reference T-RF, are calculated for all other T-RFs and the standard deviation of the T-RF sizes is calculated for all alignment bins. All common T-RFs are tested as references and the T-RF which results in the lowest size variation within the alignment bins, i.e. the lowest sum of the standard deviations of all alignment bins, is chosen. The chosen reference T-RF is given the same value in all replicates, the average of the original sizes, and new sizes are calculated for the other T-RFs based on their relative sizes. The procedure uses the sheet “TempWorking” for calculations and the data in the sheet “InData”. Creates a new sheet called ”ReplicateRelativeSizes” with the sizes relative to the reference T-RF and a sheet called ”NewReplicateSizes” with recalculated sizes based on the average size of the reference T-RF.

#### Macro: F2_AlignReplicatesWithRelativeSizes

This procedure aligns the replicate profiles with new T-RF sizes using the moving average procedure described above for the alignment of the original replicate profiles**.** The only data used here is the size data, not peak height or area data. The procedure uses the sheets “TempWorking” and “TempInData” for calculations, the data in the sheet “InData” and the parameters in the sheet “Input”. Creates a new sheet called ”NewReplicateSizesAligned”.

#### Macro: F3_FindAndCorrectAmbiguousAlignments

This procedure is identical to *D_FindAndCorrectAmbiguousAlignments*, described above, except that no new sheet is created and the corrections are made in the ”NewReplicateSizesAligned” sheet. The number of possibly ambiguous T-RFs and the number of corrected alignments are written in the “Input sheet”.

#### Macro: F4_CompareNewRepSizesAlWithInData

This procedure compares the alignment of the systematic shift corrected T-RF sizes with the original alignment. The procedure uses the data in the sheet “InData”, which is the original alignment, and “NewReplicateSizesAligned”, which is the new alignment. Cells in ”NewReplicateSizesAligned” that differs from “InData” are highlighted in red. The number of replicate T-RFs placed in different bins when relative sizes are used is written in the “Input sheet”.

### Generation of consensus profiles

These procedures combines the aligned replicate profiles into one consensus profile. The T-RFs are considered for the consensus profiles if they are present in at least X replicates. The parameter X must be given in the “Input” sheet. The T-RF sizes of the consensus profile are the average sizes of the T-RFs in the replicates. The peak heights and areas can be calculated as either the average or the sum of the peak heights and areas of the replicates.

#### Macro: G_RepConsensus_Average

This procedure creates consensus profiles from the replicates. A T-RF is considered if it is present in X number of replicates as specified in the “Input” sheet. The new size, height and area data is the average value of all replicates. The procedure uses the data in “InData” and creates a new sheet called ”RepConsensus”. The data in the sheet ”RepConsensus” is copied to the sheet “InData” for further analysis.

#### Macro: G_RepConsensus_Sum

This procedure creates consensus profiles from the replicates. A T-RF is considered if it is present in X number of replicates as specified in the “Input” sheet. The new size is the average value of all replicates but the height and area data is the sum of all replicates. The procedure uses the data in “InData” and creates a new sheet called ”RepConsensusSum”. The data in the sheet ”RepConsensus” is copied to the sheet “InData” for further analysis.

### Normalization of consensus profiles

There are four options for the normalization of consensus profiles. Two use the TFN procedure described above for the normalization of replicate profiles with total fluorescence defined as the sum of either all peak areas or all peak heights. The other two use the FPT threshold described above for the normalization of replicate profiles. For the FPT threshold approach the previously described procedures *B_Normalize_all_FPTAreas* and *B_Normalize_all_FPTHeights* can be used. Note that if these procedures have already been used for the normalization of replicates the sheets that were created, ”AllNormalizedFPTAreas” and ”AllNormalizedFPTHeights”, must be renamed before running the procedures.

#### Macro: H_Normalize_all_TFNAreas

This procedure normalizes all samples using the TFN procedure, defining the total fluorescence as the sum of all peak areas. The minimum allowed peak area is the minimum observed peak area among all profiles in the dataset. The procedure uses the sheet “TempWorking” for calculations, the data in the sheet “InData” and the parameters in the sheet “Input”. A new sheet called ”AllNormalizedTFNAreas” is created. The data in the sheet ”AllNormalizedTFNAreas” is copied to the sheet “InData” for further analysis.

#### Macro: H_Normalize_all_TFNHeights

This procedure normalizes all samples using the TFN procedure, defining the total fluorescence as the sum of all peak heights. The minimum allowed peak height is the defined peak detection threshold in the “Input” sheet. The procedure uses the sheet “TempWorking” for calculations, the data in the sheet “InData” and the parameters in the sheet “Input”. A new sheet called ”AllNormalizedTFNHeights” is created. The data in the sheet ”AllNormalizedTFNHeights” is copied to the sheet “InData” for further analysis.

### Alignment of consensus profiles

The consensus profiles are aligned using the moving average procedure as described above for the alignment of replicate profiles. The number of T-RFs and the minimum, maximum and average T-RF size is then calculated for all alignment bins. The alignment bins are also checked and bins that are possibly ambiguous are highlighted. The alignment of the T-RFs is what determines how similar the profiles are and since the purpose of a T-RFLP analysis often is to identify differences in microbial community composition between samples it is important that the alignment is done in an adequate way. Here, an alignment bin is considered ambiguous if the longest T-RF of the bin is within the alignment range of the shortest T-RF in the following alignment bin. This means that a pair-wise comparison of the samples, or an analysis of a subset of the samples, would result in a different alignment. Table 6 shows an example of an alignment bin that was classified as ambiguous. After the automatic alignment of the profiles in Table 6 sample 1, 2 and 4 were determined to share one component (one T-RF) of the bacterial community while sample 3, 5 and 6 shared another. However, as detected by the control procedure, *J_Check_AlignedConsensus*, this does not seem entirely correct. The T-RF in the profile of sample 5 is outside the alignment range of the T-RFs of the profiles of sample 1 and 2 and within the alignment range of the T-RFs in the profiles of samples 3, 4 and 6, but it was only aligned with the profiles of sample 3 and 6. Furthermore, if we would exclude the profiles of sample 2 and 5 from the analysis, the T-RFs in all remaining profiles would be aligned together and not in different bins, as in Table 6. As the alignment bins cannot be separated in a convincing unambiguous way they are classified as ambiguous. As in the correction of the alignment of replicate profiles, alignment bins may be marked as ambiguous although they are accurate (Table 7).

**Table 6:** **Example of two alignment bins classified as ambiguous**

**Samples**	**1**	**2**	**3**	**4**	**5**	**6**	**No of T-RFs**	**Min size**	**Max size**	**Mean size**	**Ambiguous**
**T-RF 1**	309.61	309.30		310.29			3	309.30	310.29	309.73	Yes
**T-RF 2**			310.31		311.18	310.46	3	310.31	311.18	310.65	Yes

The table replicates the output of the procedure “*J_Check_AlignedConsensus*”. Following the columns of the six samples, where the given numbers are the T-RF sizes in bases, are columns for the number of T-RFs, the minimum T-RF size, the maximum T-RF size and the mean T-RF size in each alignment bin. The last column is either “Yes” or “No”, indicating whether the alignment bin is ambiguous or not.

**Table 7:** **Examples of correct alignments that are classified as ambiguous**

**Samples**	**1**	**2**	**3**	**4**	**No of T-RFs**	**Min size**	**Max size**	**Mean size**	**Ambiguous**
**T-RF 1**	162.99	163.30	163.32	163.03	4	162.99	163.32	163.16	Yes
**T-RF 2**	164.10	164.88	164.65	164.28	4	164.10	164.88	164.48	Yes
**T-RF 3**	166.18	0	0	166.95	2	166.18	166.95	166.57	Yes
**T-RF 4**	167.97	168.25	168.22	167.77	4	167.77	168.25	168.05	Yes

The table replicates the output of the procedure “*J_Check_AlignedConsensus*”. Following the columns of the six samples, where the given numbers are the T-RF sizes in bases, are columns for the number of T-RFs, the minimum T-RF size, the maximum T-RF size and the mean T-RF size in each alignment bin. The last column is either “Yes” or “No”, indicating whether the alignment bin is ambiguous or not.

#### Macro: I_Align_consensus_profiles

This procedure aligns the consensus profiles using the moving average procedure described above for the alignment of replicate profiles. The procedure uses the sheet “TempWorking” for calculations, the data in the sheet “InData” and the parameters in the sheet “Input”. A new sheet called ”AlignedConsensus” is created. The number of alignment bins is written in the “Input sheet”. The data in the sheet ”AlignedConsensus” is copied to the sheet “InData” for further analysis.

#### Macro: J_Check_AlignedConsensus

This procedure checks the alignment, counts fragments and calculates minimum, maximum and average sizes. Alignment bins that are considered ambiguous are marked as such in the sheet “AlignedConsensus”. The number of possibly ambiguous T-RFs is written in the “Input sheet”. The data in the sheet ”AlignedConsensus” is copied to the sheet “InData” for further analysis.

### Detection and correction of systematic differences in size estimation between consensus profiles

As in the analysis of the replicate profiles, all consensus profiles are compared pair-wise to detect if there are systematic differences in the estimation of T-RF sizes. The systematic shift is corrected for, new T-RF sizes are calculated and the profiles are re-aligned. The calculation of new T-RF sizes and re-alignment of the profiles is done for all profiles, regardless if there was a systematic shift towards any other profile or not. The comparison with the original alignment must be done manually. If the alignment of the consensus profiles should be corrected after comparison with the alignment of shift-corrected T-RFs the correction must be done manually, changing the size, height and area data.

#### Macro: K_CheckConsensusSystematicShifts

Pairwise comparisons are made between all consensus profiles. If all T-RFs in one of the profiles are shorter than all T-RFs in the other, there is a systematic shift in size estimation between the two. The procedure uses the data from the sheet “InData” and “Input” and creates new sheets called ”ConsRepSystematicShiftCheck” and ”ConsRepSystematicShiftMatrix”. Five columns after the last sample in the “InData” sheet there must be a column with information about which alignment bins that should be disregarded for the analysis, i.e. if the alignment is correct or ambiguous (this is the format of the “AlignedConsensus” sheet after the procedure *J_Check_AlignedConsensus*). The value should be ”Yes” for ambiguous bins and ”No” for correct bins. In ”ConsRepSystematicShiftCheck” the result of all T-RF size comparisons are given and in ”ConsRepSystematicShiftMatrix” there is a matrix showing which profiles that display a systematic shift towards one another.

#### Macro: L_Shift_corrected_consensus_sizes

This procedure calls the procedures L1 to L3 for calculation and analysis of systematic shift corrected T-RF sizes.

#### Macro: L1_RelativeConsensusSizes

This procedure calculates new, relative, T-RF sizes. A T-RF common to all consensus profiles is chosen as a reference fragment and the size of the T-RF is set to 0 for all profiles. Relative sizes, i.e. the difference in size between a T-RF and the reference T-RF, are calculated for all other T-RFs and the standard deviation of the T-RF sizes is calculated for all alignment bins. All common T-RFs are tested as references and the T-RF which results in the lowest size variation within the alignment bins, i.e. the lowest sum of the standard deviations of all alignment bins, is chosen. The chosen reference T-RF is given the same value in all profiles, the average of the original sizes, and new sizes are calculated for the other T-RFs based on their relative sizes. The procedure uses the sheets “TempWorking” for calculations and the data in the sheet “InData”. Three new sheets are created: ”ConsRelativeSizesStDevs”, which lists all reference fragments and the corresponding standard deviations, ”ConsensusRelativeSizes”, which contains the sizes relative to the reference T-RF and ”NewReplicateSizes”, which include the recalculated sizes based on the average size of the reference T-RF.

#### Macro: L2_AlignConsensusRelativeSizes

This procedure aligns the consensus profiles with the new T-RF sizes using the moving average procedure described above for the alignment of replicate profiles**.** The procedure only handles size data and do not align or use peak height or area data. The procedure uses the sheets “TempWorking” and “TempInData” for calculations, the data in the sheet “InData” and the parameters in the sheet “Input”. A new sheet called ”AlignedConsShiftCorrSizes“ is created.

#### Macro: L3_Check_AlignedConsSystShiftCorrSizes

This procedure checks the alignment, counts fragments and calculates minimum, maximum and average sizes. Alignment bins that are considered ambiguous are marked as such in the sheet ”AlignedConsShiftCorrSizes“. The number of possibly ambiguous T-RFs is written in the “Input sheet”.

### Calculation of the relative abundances of T-RFs

The relative abundances of the T-RFs, instead of the absolute numbers, are often used for the analyses of the T-RF profiles. The relative abundance of a T-RF is calculated as the peak height (or area) divided by the sum of all peak heights (or areas) of the T-RF profile.

#### Macro: M_Relative_abundance_Heights

This procedure calculates the relative abundance of the T-RFs of all profiles. The total fluorescence is defined as the sum of all peak heights. The procedure uses the data from “InData” and creates a new sheet called ”RelativeAbundanceHeights”. The data in the sheet ”RelativeAbundanceHeights” is copied to the sheet “InData” for further analysis.

#### Macro: M_Relative_abundance_Areas

This procedure calculates the relative abundance of the T-RFs of all profiles. The total fluorescence is defined as the sum of all peak areas. The procedure uses the data from “InData” and creates a new sheet called ”RelativeAbundanceAreas”. The data in the sheet ”RelativeAbundanceAreas” is copied to the sheet “InData” for further analysis.

### Calculation of association coefficients

Using the relative abundance data two common association coefficients can be calculated: the Bray-Curtis distance coefficient, which takes the relative abundance of the T-RFs in consideration, and the Jaccard similarity coefficient, which put equal weight on all T-RFs, regardless of their relative abundance.

#### Macro: N_BrayCurtis_matrix

This procedure calculates the Bray-Curtis distance (as described in [18]) for all pair-wise comparisons of the T-RF profiles. The procedure uses the data in “InData” and creates a new sheet called ”BrayCurtisMatrix”. The formula for the calculation is:

$$ Bray- Curtis\kern0.5em  distance\kern0.5em  between\kern0.5em  profile\kern0.5em X\kern0.5em  and\kern0.5em Y=\frac{{\displaystyle {\sum}_{i=1}^n Abs\left({x}_i-{y}_i\right)}}{{\displaystyle {\sum}_{i=1}^n\left({x}_i+{y}_i\right)}}, $$

where n is the total number of aligned T-RFs and *x*_*i*_ and *y*_*i*_ is the relative abundance of T-RF *i* in profile *X* and *Y*, respectively.

#### Macro: O_JaccardCoeff_matrix

This procedure calculates the Jaccard similarity coefficient (as described in [18]) for all pair-wise comparisons of the T-RF profiles. The procedure uses the data in “InData” and creates a new sheet called ”JaccardCoeffMatrix”. The formula for the calculation is:

$$ Jaccard\  similarity\  between\  profile\ X\  and\ Y=\frac{Number\  of\  aligned\ T-RFs\  present\  in\  both\  profile\ X\  and\  profile\ Y}{Total\  number\  of\  aligned\ T-RFs\  in\  profile s\ X\  and\ Y} $$

### Calculation of diversity index

Using the relative abundance data the widely used Shannon diversity index can be calculated. Note that the diversity index should only be used for comparison with other profiles in the same dataset that have been analyzed the same way. The number of T-RFs in the profile is highly dependent on the applied peak detection threshold, the normalization and how the consensus profile was generated. Therefore, the diversity index should not be used as an absolute value and should not be compared with diversity indices of samples from other analyses.

#### Macro: P_ShannonIndex

This procedure calculates the Shannon diversity index (as described in [18]) for all profiles. The procedure uses the data in “InData” and creates a new sheet called ”Shannon Index”. The formula for the calculation is:

$Shannon\kern0.75em  index\  of\  profile\ X=-{\displaystyle {\sum}_{i=1}^n\left({x}_i\ast \kern0.6em  \log \kern0.5em \left({x}_i\right)\right),}$ where n is the total number of aligned T-RFs and *x*_*i*_ is the relative abundance of T-RF *i* in profile *X*. The base of the logarithm is 10.

### Example dataset

The dataset used here was taken from a study of bacterial dynamics in a wastewater treatment plant (WWTP) and is provided in Additional file 2.

### Sample collection and DNA extraction

Samples were collected at the end of the aerated basins at the Rya WWTP, a WWTP treating both industrial and municipal wastewater [19]. Permission to enter the Rya WWTP and to collect activated sludge samples were granted by Gryaab AB (owner and operator of the WWTP). 50 mL of sample were centrifuged and the resulting pellet was stored at −20°C within 1.5 h from collection. DNA was extracted using Power Soil DNA Extraction Kit (MoBio Laboratories). The frozen sludge pellets were thawed, 15 mL sterile water were added and the samples were homogenized by 6 min of mixing in a BagMixer 100 MiniMix (Interscience). Water was removed by centrifugation and DNA was extracted from 0.25 g of homogenized sludge pellet according to the manufacturer’s instructions.

### PCR

16S rRNA genes were amplified using HotStarTaqPlus PCR kit (Qiagen) according to the manufacturer’s instructions. The *Bacteria*-specific primer pair 63 F (CAGGCCTAACACATGCAAGTC) and M1387R (GGGCGGWGTGTACAAGRC) were used. The primer pair was based on the sequences 63F and 1387R [20], which were previously evaluated using strains of all major bacterial groups, including Gram-positive bacteria, and was found to be more successful than the commonly used primer pair 27F&1392R [21]. The primer 1387R has a mismatch for some bacterial sequences at position 1388 [20] and was therefore modified. The forward primer 63F was labeled at the 5’-end with the fluorescent dye 6 – carboxyfluorescein. PCR reactions were carried out in the provided PCR buffer with 0.5 U HotStarTaqPlus, 200 μM dNTP mix, 0.1 μM of each primer and 2–5 ng DNA. The cycle profiles had an initial 5 min at 95°C for Taq polymerase activation followed by 35 cycles of denaturation at 94°C for 1 min, annealing at 60°C for 30 s and elongation at 72°C for 1 min. The reactions were ended with a final elongation step at 72°C for 7 min.

### T-RFLP

The PCR products were purified using the Agencourt AMPure system (Beckman Coulter) and digested with 10 units of restriction enzyme *Hha*I or *Rsa*I (New England Biolabs) in the manufacturer’s provided buffers 4 or 1, respectively. Digestion was carried out at 37°C for at least 16 hours and the restriction digests were purified using the Agencourt AMPure system. For each reaction, 2 μl purified restriction fragments were added to 6.7 μl formamide and 0.3 μl of the size standard LIZ1200 (Applied Biosystems). The fragments were analyzed by capillary gel electrophoresis (3730 DNA Analyzer, Applied Biosystems) using a 20s injection time, a 2.0 kV injection voltage and a 9 kV run voltage. The software GeneMapper (Applied Biosystems) was used to quantify the electropherogram data and to generate the T-RF profiles. The settings used for the raw data processing in GeneMapper was a peak detection threshold of 50, default peak detection settings and the Local Southern size calling algorithm. Data for presence/absence, size, peak height and peak area was retrieved for all T-RFs in all samples using the GeneMapper Report Manager.

### Data analysis

The dataset was prepared with analysis range 50 to 1020 bases and peak detection threshold 50 and 100, separately. For the data generated with peak detection threshold 100, sample duplicates were normalized using all five normalization methods. After normalization with TFN-heights, sample duplicates were aligned, checked for systematic differences in T-RF size estimation, and used to generate consensus profiles, considering T-RFs present in both or only one of the two replicate profiles. The height and area of the consensus T-RFs were calculated as the average of the heights and areas of the replicate profiles. The consensus profiles with only T-RFs present in both replicates were then normalized using all four methods. The consensus profiles normalized with the TFN-heights procedure were used for calculations of the relative abundances of the T-RFs and for calculation of association coefficients and diversity index. As a comparison a matrix of Jaccard coefficients was also calculated for the dataset treated without alignment correction and without normalization. The example data was also analyzed using *T-REX* with the following settings: Noise filtering was performed using the procedure described by Abdo *et al*. [7] for all samples based on peak heights. The standard deviation multiplier was set to 1. The T-RFs were aligned using the *T-Align* method [10] with a clustering threshold of 1, allowing at most one peak per plot in each T-RF. After alignment a data matrix was constructed using the average peak height data of the replicates. The resulting peak heights were relativized within samples. The data was exported and the Jaccard coefficients were calculated using the *Tools for T-RFLP data analysis*.

## Results and discussion

The functionality of the provided procedures in the *Tools for T-RFLP data analysis* was compared with the available software *T-Align* [10], *T-REX* [17] and the script package *T-RFLP Stats* [7]. A dataset of 76 T-RF profiles from 38 activated sludge samples were used to illustrate the procedures (Additional file 2).

### Data preparation

Using the *Tools for T-RFLP data analysis* peak detection thresholds of the users choice can be applied before any other analysis is carried out. At this stage the user also specifies the analysis range, i.e. restricting the analysis to only include T-RFs of lengths between a minimum and maximum value. Neither of these two options is available in any of the other three programs. Instead of using a fixed baseline threshold to remove noise and false peaks, both *T-REX* and *T-RFLP Stats* use the noise filtering method presented by Abdo *et al*. [7].

With an applied peak detection threshold of 50, as for example Culman *et al*. [22], the average number of T-RFs of the 76 profiles in the example dataset were 32 ± 11. Increasing the threshold to 100, as Osborn *et al*. [5], decreased the average number of T-RFs to 20 ± 7. The threshold level should be set high enough to exclude noise peaks from the analysis but how high the threshold needs to be to ensure this may vary between different analytical platforms and analyses. A wide range of peak detection thresholds can be found in the literature, from as low as 25 [6] to as high as 200 [22].

### Normalization of replicate profiles

Neither *T-Align, T-REX* or *T-RFLP Stats* include any normalization method. The *Tools for T-RFLP data analysis* provide five different methods for the normalization of the replicate profiles. Having various available options for normalization allows for comparisons of the different methods.

**Figure 2:**
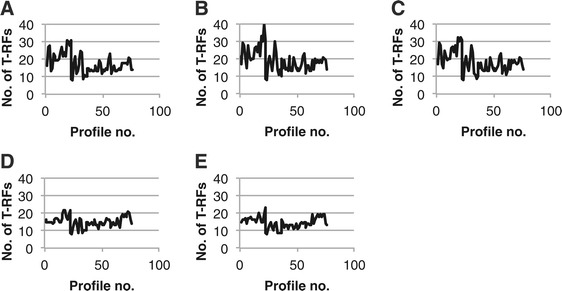
**Number of remaining T-RFs after normalization of replicate profiles.** Each of 38 replicate profile pairs was normalized using the procedures TFN-heights **(panel A)**, TFN-Areas **(panel B)**, TFN-Areas-LT **(panel C)**, FPT-heights **(panel D)** and FPT-areas **(panel E)**. As a result of normalization, T-RFs are removed from the profiles. The figures show the number of T-RFs in each profile after normalization.

In the example dataset, the number of remaining T-RFs in the profiles after normalization was different for all five methods (Figure 2). The methods included the previously published methods TFN-heights [23], TFN-areas-LT (local threshold) [12] and FPT-areas [14] as well as the variants TFN-areas and FPT-heights.

### Alignment of replicate profiles

The *Tools for T-RFLP data analysis* implements the same alignment procedure as *T-Align* and *T-REX,* with the exception that the user can specify two of the parameters for the alignment instead of just one. This enables the user to adjust the algorithm so that it best suits the properties of the data, i.e. the observed range of variation in T-RF size estimations. Another improvement of the alignment procedure is the correction of the alignment. The alignment produced by the algorithm used in *T-Align* does not always align the T-RFs correctly (Table 4, Automatic alignment) and in *Tools for T-RFLP data analysis* an option to automatically correct the alignment of replicate profiles is provided (Table 4, Corrected alignment).

In the example dataset four alignment bins had to be corrected after alignment of the replicate T-RF profiles.

### Detection and correction of systematic differences in size estimation between replicates

The *Tools for T-RFLP data analysis* also implements an additional approach for evaluation and correction of the alignment of the T-RFs. This is not provided by any of the other available programs. If the number of replicates is only two, the automatic correction of the alignment is enough, but for higher number of replicates the procedures can be of value, as for the alignment of consensus profiles (see discussion below).

Although the alignment of the replicate profiles in the example dataset already was corrected, the data was checked for systematic differences in T-RF size estimations. Five replicate pairs showed a systematic difference in T-RF lengths. However, the alignment obtained after adjusting for the systematic shift was exactly the same as the original corrected alignment.

### Generation of consensus profiles

In *T-Align* the default setting for generation of consensus profiles is to only consider T-RFs present in all replicates and the average value of the peak heights and areas corresponding to those T-RFs are then calculated. In *T-REX* consensus profiles are generated by averaging the values of all T-RFs in the replicates, independent of the number of replicates that the T-RFs are present in. The *Tools for T-RFLP data analysis* allows the user to specify how the consensus profiles are generated: which T-RFs to consider and if the average or the sum of the peak height and area values should be used. The option to use the sum of the peak heights and areas of replicates could be used to emulate the effect of pooling replicates before the loading on the gel.

**Figure 3:**
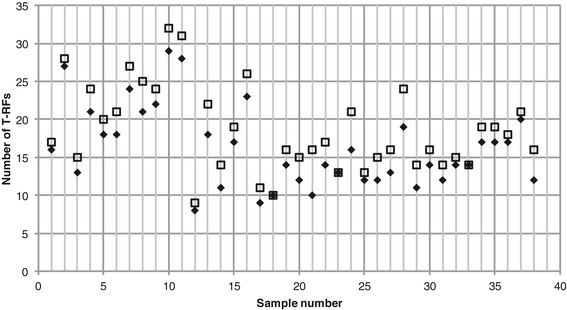
**Number of T-RFs in the consensus profiles.** T-RFs were included in the consensus profiles if they were present in one (squares) or both (diamonds) of the two replicates.

Consensus profiles generated by only considering T-RFs that were present in both of the two replicates, as suggested by Dunbar *et al*. [6], had fewer T-RFs than the consensus profiles generated considering all T-RFs in both replicate profiles, as in the program *T-REX* (Figure 3).

### Normalization of the consensus profiles

There are four options for the normalization of consensus profiles. As stated before, neither *T-Align, T-REX* or *T-RFLP Stats* include any normalization method.

**Figure 4:**
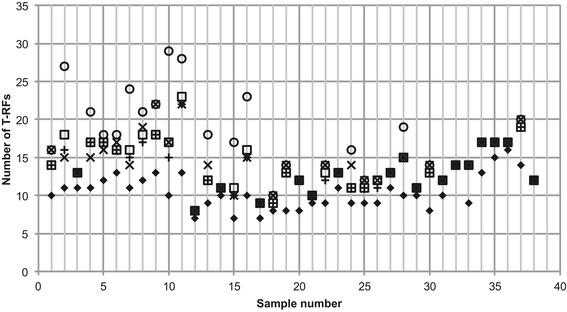
**Number of remaining T-RFs after normalization of consensus profiles.** The consensus profiles were normalized using the procedures TFN-heights (diamonds), TFN-Areas (empty squares), FPT-heights (X) and FPT-areas (+).

All four available normalization methods were applied in the analysis of the example dataset. The TFN-heights method [24] reduced the number of T-RFs the most (Figure 4).

### Alignment of the consensus profiles

The consensus profiles are aligned the same way as the replicate profiles. There is no correction procedure for the alignment of the consensus profiles, but a control function is provided where potentially erroneous alignment bins are classified as ambiguous and highlighted.

**Figure 5:**
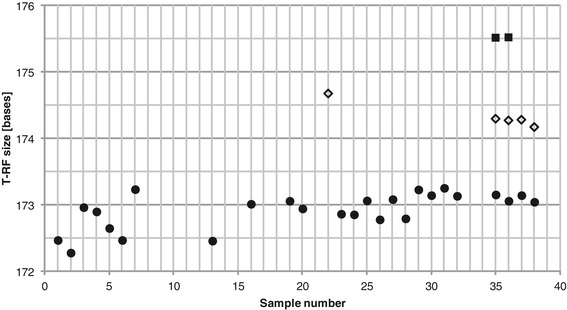
**Example of correct alignment bins classified as ambiguous.** The symbols represent the alignment bins created in the automatic alignment procedure.

**Figure 6:**
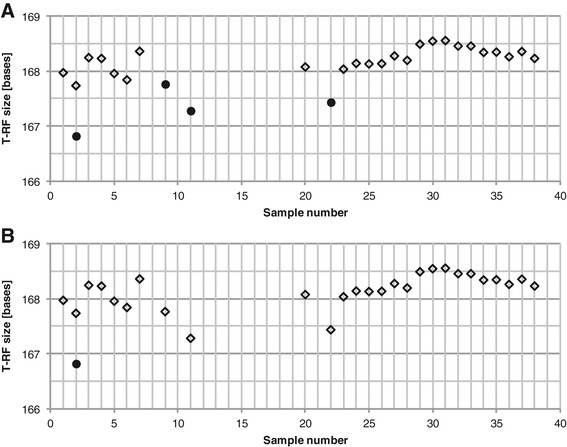
**Example of correction of an ambiguous alignment bin.** Alignment bins created in the automatic alignment **(panel A)** and after manual correction **(panel B)**. The symbols represent the two alignment bins. Only sample 2 had T-RFs in both alignment bins. The correction was motivated by the observation that the T-RFs in the profiles of all other samples were closer in size to the longer T-RF than the shorter T-RF in sample 2.

**Figure 7:**
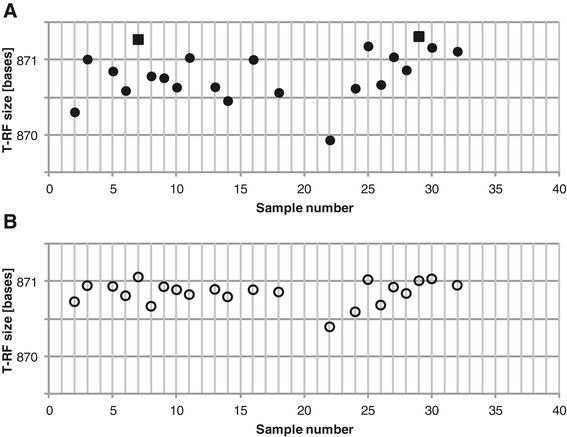
**Example of systematic shift correction.** Alignment of original T-RF sizes **(panel A)** and systematic shift corrected T-RF sizes **(panel B)**. The symbols represent the alignment bins created in the automatic alignment.

In the example dataset the alignment resulted in 30 alignment bins of which 17 were classified as ambiguous. By inspection, 6 of the ambiguous alignment bins were determined to be correct (see example in Figure 5) and 2 were manually corrected (see example in Figure 6). However, 9 alignment bins still remained ambiguous (see example in Figure 7).

### Detection and correction of systematic differences in size estimation between consensus profiles

The detection and correction of systematic differences in T-RF size estimation between consensus profiles is done the same way as for the replicate profiles. There are generally more systematic differences between the consensus profiles than between the replicate profiles and consequently the differences between the original and the systematic shift-corrected alignment are often greater for the consensus profiles than for the replicate profiles.

By adjusting for the systematic differences in size estimation, the alignment could be resolved for six of the nine remaining ambiguous alignment bins (see example in Figure 7) in the example dataset.

### Calculation of the relative abundances of T-RFs

The relative abundances of the T-RFs can be calculated using either peak heights or areas. This is possible both in *T-REX* and in *Tools for T-RFLP data analysis*.

### Calculation of association coefficients and diversity index

Neither one of the three available programs (*T-REX*, *T-Align* and *T-RFLP Stats*) provides an option to compare the T-RF profiles by calculating association coefficients or diversity indices. In the *Tools for T-RFLP data analysis* there are two association coefficients available: the Jaccard similarity coefficient and the Bray-Curtis distance coefficient, and one diversity index: the Shannon diversity index. There are other programs dedicated to statistical analysis which provide a wide range of association coefficients and diversity indices, for example *PAST* [24]. However, for convenience these two common association coefficients and one diversity index were included in the *Tools for T-RFLP data analysis*.

**Figure 8:**
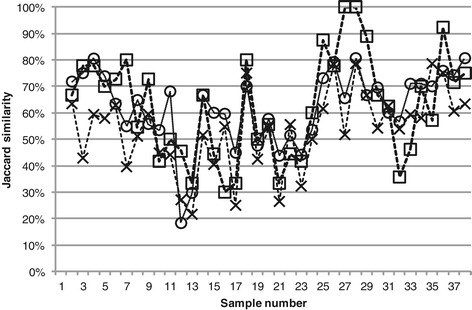
**Jaccard similarity between all profiles and the profile of the first sample.** The data was treated in three different ways. X: PDT 50, No normalization, no alignment correction. Squares: PDT 100, TFN-heights, normalization of both replicate and consensus profiles. Circles: *T-REX* based on peak heights. In all three treatments consensus profiles were generated only considering T-RFs present in both replicates.

The Jaccard similarities between all profiles and the profile of the first sample in the dataset were calculated for three different treatments of the example dataset (Figure 8). Figure 8 clearly shows that how T-RFLP data is treated has an impact on the final comparisons of community composition. To evaluate which treatment approaches that are the most accurate the *Tools for T-RFLP data analysis* presented here can be of great use.

## Conclusions

The *Tools for T-RFLP data analysis* provide easily adjustable and expandable tools for the analysis of T-RFLP data. It enables evaluations of the impact of different data treatment methods on the final outcome of sample comparisons, combinations of different treatment methods and provides tools to evaluate the accuracy of the alignment of T-RFs. The *Tools for T-RFLP data analysis* is implemented in Microsoft Excel and therefore requires a purchased license for use. Although this is a drawback, it is likely to be a both familiar and accessible environment for many researchers.

## Availability and requirements

**Project name:** Tools for T-RFLP data analysis

**Project home page:**http://sourceforge.net/projects/toolsfortrflp

**Operating system(s):** Microsoft Office

**Programming language:** Visual Basic for Applications (VBA)

**License:** GNU Lesser General Public License

## Availability of supporting data

The *Tools for T-RFLP data analysis* template is provided in Additional file 1. The dataset used to illustrate the procedures is provided in Additional file 2.

## Additional files

Additional file 1:
**Tools for T-RFLP data analysis.**
Additional file 2:
**Supporting data.**

## Abbreviations

FPT: Fixed percentage threshold
TF: Total fluorescence
TFN: Total fluorescence normalization
T-RF: Terminal restriction fragment
T-RFLP: Terminal restriction fragment length polymorphism
